# Evolution of elliptical SAXS patterns in aligned systems

**DOI:** 10.1107/S1600576724004503

**Published:** 2024-07-11

**Authors:** N. Sanjeeva Murthy, David T. Grubb

**Affiliations:** a Rutgers University, New Brunswick, New JerseyUSA; b Cornell University Ithaca New YorkUSA; Tohoku University, Japan

**Keywords:** aligned objects, lamellar arrays, semicrystalline polymers, small-angle X-ray scattering, elliptical traces

## Abstract

Small-angle X-ray and neutron scattering (SAXS and SANS) patterns from certain semicrystalline polymers and liquid crystals contain discrete reflections from ordered assemblies and central diffuse scattering (CDS) from uncorrelated structures. Systems with imperfectly ordered lamellar structures aligned by stretching or by a magnetic field produce four distinct SAXS patterns: two-point ‘banana’, four-point pattern, four-point ‘eyebrow’ and four-point ‘butterfly’. The peak intensities of the reflections lie not on a layer line, or the arc of a circle, but on an elliptical trajectory. Modeling shows that randomly placed lamellar stacks modified by chain slip and stack rotation or interlamellar shear can create these forms. On deformation, the isotropic CDS becomes an equatorial streak with an oval, diamond or two-bladed propeller shape, which can be analyzed by separation into isotropic and oriented components. The streak has elliptical intensity contours, a natural consequence of the imperfect alignment of the elongated scattering objects. Both equatorial streaks and two- and four-point reflections can be fitted in elliptical coordinates with relatively few parameters. Equatorial streaks can be analyzed to obtain the size and orientation of voids, fibrils or surfaces. Analyses of the lamellar reflection yield lamellar spacing, stack orientation (interlamellar shear) angle α and chain slip angle ϕ, as well as the size distribution of the lamellar stacks. Currently available computational tools allow these microstructural parameters to be rapidly refined.

## Abstract 2

Full pattern analyses of small-angle X-ray and neutron scattering data with both discrete reflections and central diffuse scattering are presented. Data with both equatorial streaks and two- and four-point reflections can be fitted in elliptical coordinates with relatively few parameters.

## 1. Introduction

Nanostructures are widely used as building blocks in nature and in the laboratory to produce optimally designed materials with the desired properties. These building blocks are hierarchically organized through self-assembly in natural materials to enhance strength and stiffness, *e.g.* in bones, abalone shells, dental tissue, tendons and hair (Espinosa *et al.*, 2009 ▸), and by thermal processing, *e.g.* spinning, drawing and 3D printing, in synthetic polymers (Baer *et al.*, 1987 ▸). Supramol­ecular assemblies of these building blocks are used in biomedical applications such as controlled-release devices (Liang *et al.*, 2022 ▸; Hedegaard & Mata, 2020 ▸). Rigid crystalline lamellae and softer non-crystalline layers, ∼100 nm wide and with alternating ∼25 nm periodicity, are the building blocks in many semicrystalline polymers. Characterization of structural features in such assemblies is important for understanding the influence of structure on macroscopic physical properties, and to achieve desired properties such as a favorable combination of stiffness and toughness. These structure–property correlations need to be established on a large scale to take advantage of machine learning (ML) and other artificial intelligence (AI) tools that are now under development (Beltran-Villegas *et al.*, 2019 ▸). Small-angle X-ray scattering (SAXS), which can provide the necessary structural information at nano-length scales (1–100 nm), is the tool of choice for such high-throughput measurements (Lombardo *et al.*, 2020 ▸).

The methods for deriving structural information from SAXS and the related technique of small-angle neutron scattering (SANS) are quite different from those used in wide-angle X-ray scattering (WAXS), primarily because of the completely different characteristics of the two types of data. In WAXS, there may be hundreds or thousands of reflections depending on the size of the unit cell (larger size allows more reflections) and the extent of long-range order (higher order produces more reflections). Crystallographic methods that derive the structure use the ‘integrated intensities’ of these reflections. This is not feasible in SAXS where there are, at most, a handful of reflections, and more often just one or two diffuse spots including the one centered on the origin. The ‘intensity variations’ within these spots are used to determine parameters related to the structure. One example of this is the determination of the radius of gyration of the scattering object by Guinier analysis of the intensity variation of the central diffuse spot (Guinier & Fournet, 1955 ▸). Similar analyses of the discrete patterns yield information about some general features of the structures such as size, interplanar spacing and orientation distribution.

More recently, 3D and domain structures have been determined by *ab initio* modeling from 1D SAXS patterns from dilute solutions (Volkov & Svergun, 2003 ▸; Svergun & Koch, 2003 ▸). Similar comprehensive analysis of patterns from solid materials is lacking. The limited progress made since their first discussion in early literature (Alexander, 1969 ▸) can be typically found in book chapters (Saldivar-Guerra & Vivaldo-Lima, 2013 ▸) and as part of the detailed analysis of specific samples such as nylon 6 (Zheng *et al.*, 1989 ▸; Murthy *et al.*, 1996 ▸, Murthy & Grubb, 2002 ▸), poly(ethyl­ene terephthal­ate) (Rule *et al.*, 1995 ▸; Murthy *et al.*, 1998 ▸; Murthy & Grubb, 2003 ▸), polyurethanes (Koerner *et al.*, 2008 ▸), polypropyl­ene (Fischer *et al.*, 2010 ▸), silk (Yang *et al.*, 1997 ▸), flax (Astley & Donald, 2001 ▸) and liquid crystals (Chakraborty *et al.*, 2013 ▸). We recently demonstrated a method in which, starting from plausible models, the model parameters are iteratively improved by comparing the simulated pattern with the observed pattern until there is a reasonable agreement between the two (Grubb *et al.*, 2021 ▸).

In this paper, after a discussion of the features in the full SAXS pattern and their relation to the structure, structural models that are consistent with the data will be presented. This will be followed by a proposal to parameterize the SAXS patterns in the solid state to rapidly quantify the structural features without relying on the description of the underlying structure. The goal is to show that the approach is more generally applicable. These procedures can be automated to featurize and classify the large number of patterns for use in ML and AI methods.

## 2. Features in SAXS patterns

**Figure 1:**
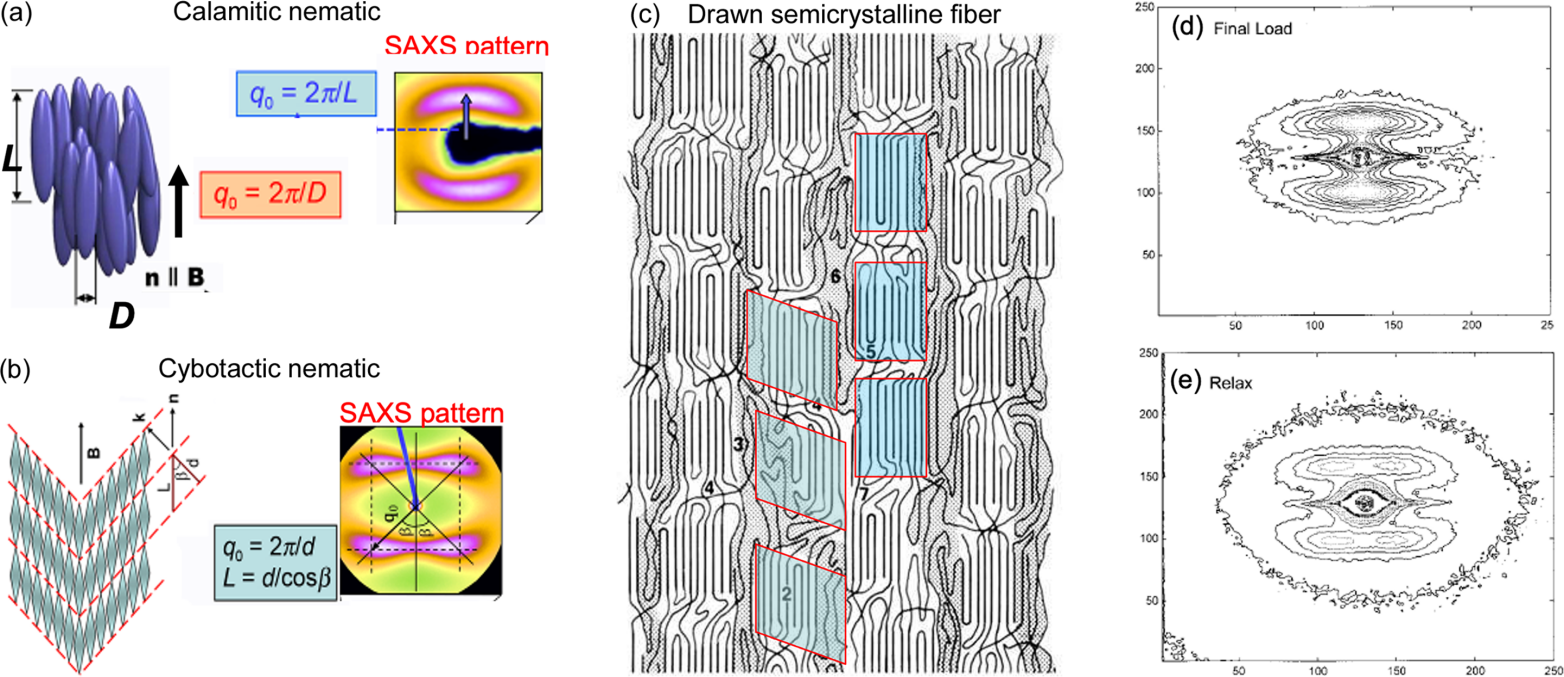
Structures and their corresponding SAXS patterns. (*a*) Two-point ‘banana’ pattern from nematic layers, adopted from Dingemans *et al.* (2013 ▸) with the permission of the publisher Taylor & Francis Ltd. (*b*) Four-point pattern from cybotactic nematic layers (Dingemans *et al.*, 2013 ▸). (*c*) Schematic of a fiber with both tilted and un-tilted lamellae (Murthy *et al.*, 1990 ▸). (*d*) Two-point pattern from an un-tilted lamellar stack. (*e*) Four-point pattern from a tilted lamellar stack. Patterns in (*d*) and (*e*) are from nylon-6 fibers (Murthy & Grubb, 2002 ▸).

SAXS patterns typically consist of discrete reflections from long-range order and central diffuse scattering (CDS) from uncorrelated structures (Fig. 1 ▸). Lamellar structures produce two apparently distinct SAXS patterns, a ‘two-point’ [Figs. 1 ▸(*a*) and 1 ▸(*d*)] and a ‘four-point’ [Figs. 1 ▸(*b*) and 1 ▸(*e*)]. When rod-like molecules in liquid crystalline phases are aligned by a magnetic field or shear, nematic and smectic-A phases produce a two-point pattern while smectic-C phases give a four-point pattern with an angle ϕ between the reflections. The splitting of each of the reflections in the two-point pattern that gives rise to the four-point pattern is caused by the lamellar normal being tilted away from the molecular axis. In some cases when the tilt is small, the reflections overlap giving the appearance of a two-point pattern, so one cannot say that a two-point pattern means no tilt. The radial position of these reflections corresponds to a lamellar spacing.

**Figure 2:**
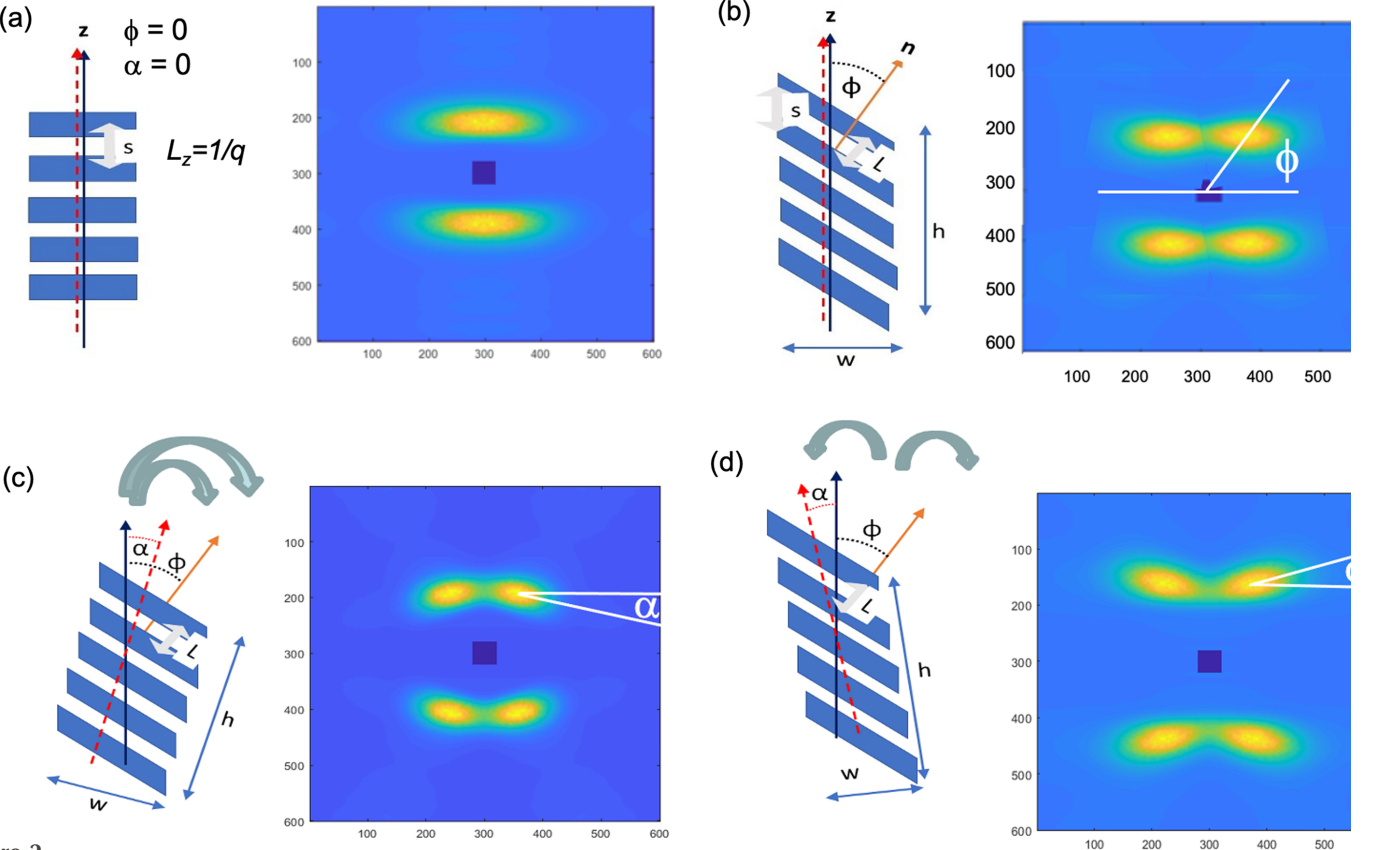
Schemes for simulating the lamellar reflections (Murthy *et al.*, 2021 ▸). Four classes of SAS lamellar arrangements and the corresponding patterns are shown: (*a*) two-point banana, (*b*) four-point, (*c*) four-point eyebrow, (*d*) four-point butterfly.

Similar two- and four-point patterns are observed in semicrystalline polymers. In polymers that crystallize as micelles or as folded-chain lamellae, there are crystalline lamellae alternating with amorphous domains [Fig. 1 ▸(*c*)] (Murthy *et al.*, 1990 ▸). Fig. 1 ▸(*d*) shows a two-point bar pattern from un-tilted lamellae in undrawn fibers. Fig. 1 ▸(*e*) shows a four-point pattern arising from tilted lamellae in drawn fibers caused by chain slip [Figs. 2 ▸(*a*) and 2 ▸(*b*)].

CDS usually arises from voids, particles, surfaces and interfaces. CDS could be in the form of circular or oval scattering, or in the form of an equatorial streak in stretched samples. This CDS is seen along with the discrete (lamellar) reflections in Figs. 1 ▸(*d*) and 1 ▸(*e*). The near-isotropic CDS can be attributed to large isotropic domains including voids, and the streak to needle-shaped voids and fibrils, interfaces, or the sample surface (Grubb & Murthy, 2010 ▸). The CDS can be analyzed to obtain the size and orientation of voids, fibrils or surfaces (Wang *et al.*, 2012 ▸) (see Section 4.2) and to understand the structural changes during deformation.

## 3. Structural models and the simulation of small-angle patterns

### 3.1. Lamellar reflections

SAXS patterns of a lamellar structure can be simulated from models of lamellar stacks constructed using the scheme in Fig. 2 ▸ with the following parameters. (1) The lamellar spacing which moves the spot along the radial direction. (2) The tilt of the lamellae which determines the separation of the spots along the azimuthal direction. (3) The rotation of the stacks which determines how much the spot rotates around its center. When the stacks rotate, either because of lamellar slip or by whole-body rotation, the reflections rotate around their center by an angle α; when the stacks rotate in the same direction as the tilt of the lamellae, an eyebrow pattern occurs; when the stacks rotate in the opposite direction to the tilt, the result is a butterfly pattern (Hay & Keller, 1967 ▸; Cowking *et al.*, 1968 ▸; Pope & Keller, 1975 ▸; Grubb *et al.*, 2021 ▸). Note that the lamellar rotation may occur by lamellar slip, which preserves the alignment of the chains along the *z* axis, or by whole-body rotation, which does not. Two more parameters are required to determine the shape of the spot. (4) The width of the stack determines the spreading of the reflection along the equatorial or the *x* axis. (5) The height of the stack determines the breadth of the reflection along the meridional direction or the *z* axis. More realistic patterns can be obtained by introducing a distribution of the various parameters.

**Figure 3:**
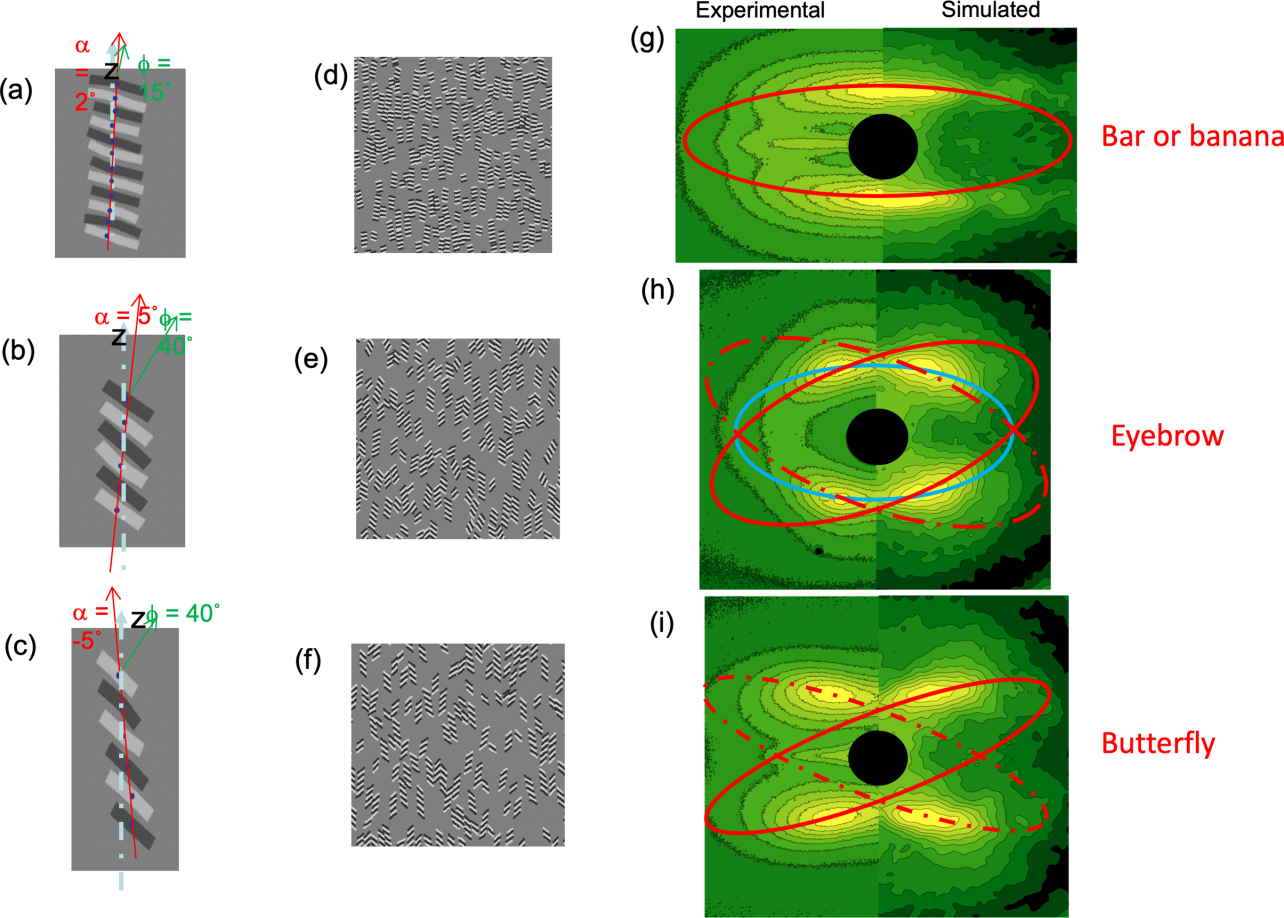
Simulation of discrete patterns by Fourier transform of model structures. The first column shows the organization of the lamellae within a stack. The second column shows the space-filled structures used to generate the diffraction patterns. The third column shows both the observed diffraction patterns (left) and the simulated patterns (right) (Grubb *et al.*, 2021 ▸; Androsch *et al.*, 2002 ▸).

**Figure 4:**
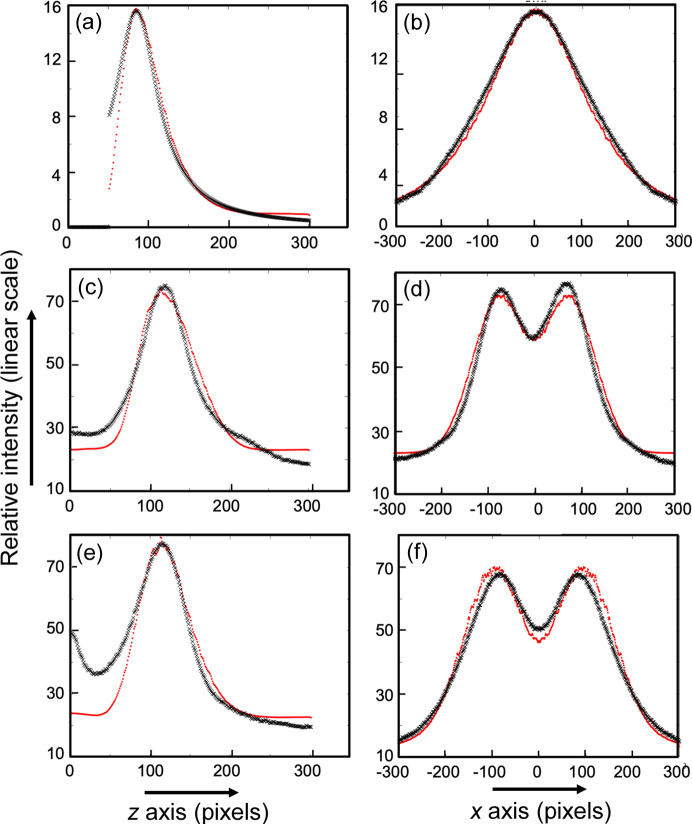
1D scans through the lamellar reflections comparing the observed (black crosses) and simulated (red dots) intensities. These figures correspond to the 2D images shown in Fig. 3 ▸. Left and right columns are slices parallel and perpendicular to the fiber axis, respectively. (*a*) and (*b*) Two-point banana-shaped pattern. (*c*) and (*d*) Four-point eyebrow pattern. (*e*) and (*f*) Four-point butterfly pattern. These are the MATLAB simulations in Figs. 6 and 7 from our earlier paper (Grubb *et al.*, 2021 ▸).

Images of lamellar stacks were generated using the model and the parameters described in the previous paragraph, and digitally packed into a box. A Fourier transform of such a box yields the diffraction pattern. Three classes of patterns obtained this way are shown in Fig. 3 ▸. The simulated patterns are in very good quantitative agreement with the observed pattern, showing that the model can capture the essential features of the structure. This can be clearly seen in the overlay of the 1D scans through the lamellar reflections in the observed and the simulated diffraction pattern (Fig. 4 ▸). From such simulations one can obtain lamellar spacing, the tilt angle of the lamellae, the rotation angle of the lamellar stack, the height and the width of the lamellar stacks, and the distribution of these parameters. These procedures have been implemented (Grubb *et al.*, 2021 ▸) in both MATLAB (The MathWorks, Nattick, MA, USA) and *Mathematica* (Wolfram Research, Champaign, Illinois, USA).

### 3.2. Central diffuse scattering

**Figure 5:**
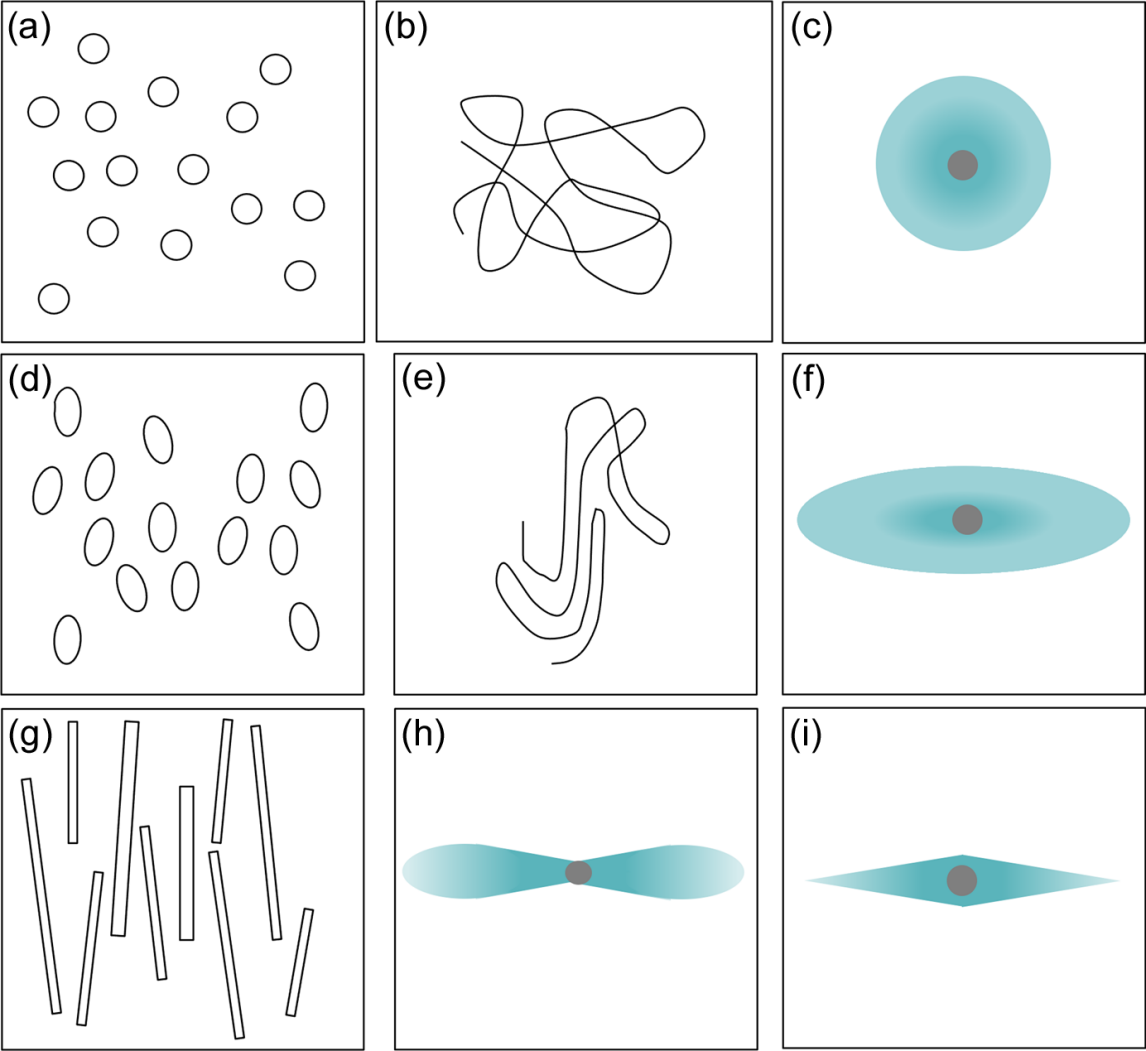
Schemes for simulating the CDS and equatorial streaks with randomly placed scattering objects. (*a*) Unoriented or spherical distribution of scattering particles. (*b*) Random chain. (*c*) Isotropic CDS. (*d*) Oriented ellipsoidal particles with some orientational correlation. (*e*) Shear-oriented polymer chain. (*f*) Oval-shaped anisotropic CDS. (*g*) Rod-like particles with preferred orientation. (*h*) Fan- and (*i*) diamond-like equatorial streaks

The central scattering can be produced using the structural models shown in Fig. 5 ▸. The shapes and arrangements of objects that give rise to the various forms of CDS and equatorial scattering fall on a continuum of structural features. A random distribution of unoriented scattering centers, voids or particles [Fig. 5 ▸(*a*)], or a random polymer chain [Fig. 5 ▸(*b*)], gives rise to isotropic CDS [Fig. 5 ▸(*c*)]. When the particles or voids are elongated but the height is of the same order of magnitude as the lateral size [*i.e.* ellipsoid-shaped, Fig. 5 ▸(*d*)], or when a polymer chain assembly is sheared or otherwise oriented [Fig. 5 ▸(*e*)], then the axial width of the reflection increases in the *x* direction such that the pattern becomes elliptical [Fig. 5 ▸(*f*)]. The scattering from well oriented rod-like particles [Fig. 5 ▸(*g*)] is an equatorial disc spread by misorientation into fan- or diamond-shaped scattering, depending on the gradient of the intensity [Figs. 5 ▸(*h*) and 5 ▸(*i*)]. With rods, the misorientation dominates to produce the fan or diamond, whereas for ellipsoids the shape transform dominates. These equatorial streaks are commonly present in Poiseuille and extension flows, and in fibrous materials containing aligned and elongated voids or surfaces.

**Figure 6:**
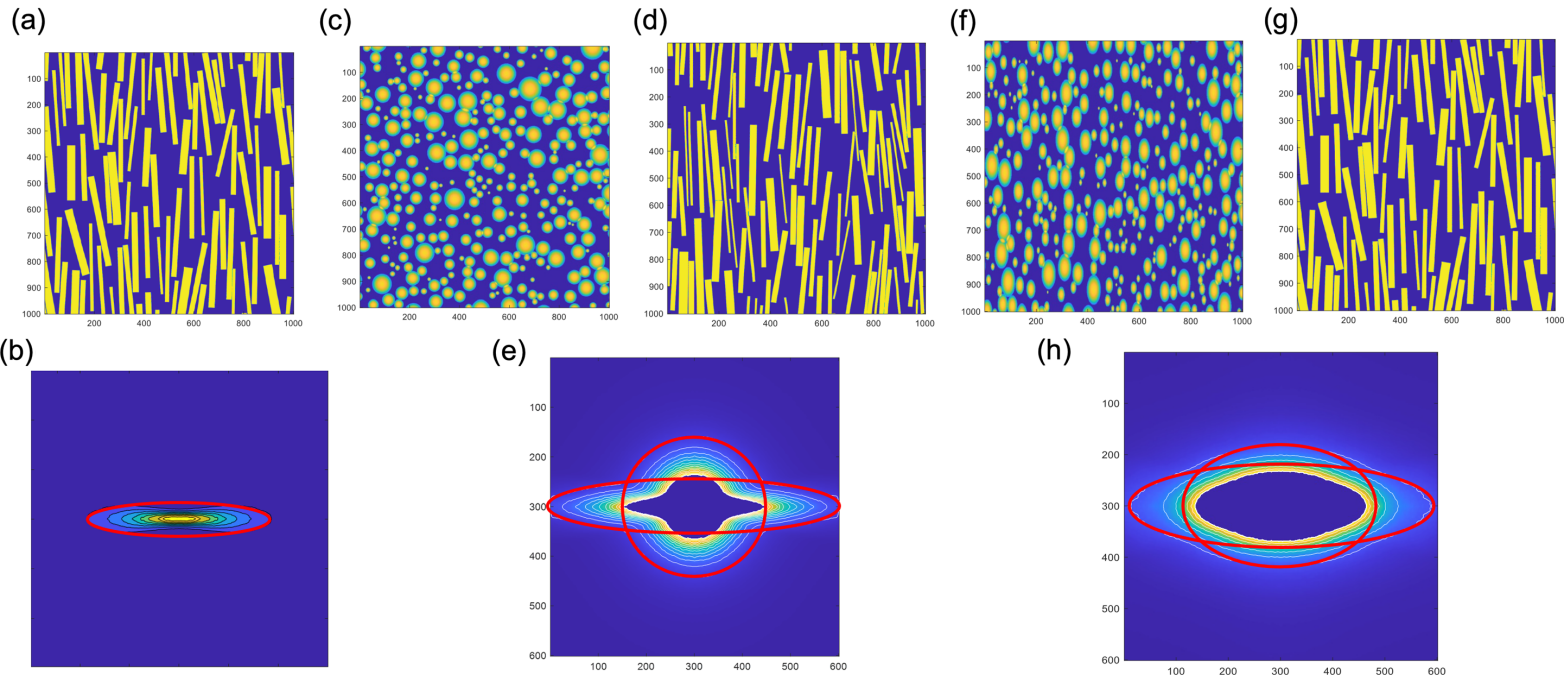
(*a*) Misorientation of the rod-like particles that gives a fan-like pattern (*b*). (*c*) and (*d*) Distribution of isotropic and rod-like particles, and (*e*) the diffraction pattern from this mixture. (*f*) and (*g*) Distribution of ellipsoid and rod-like particles, and (*h*) the diffraction pattern from this mixture.

Some CDS patterns, including equatorial scattering, were simulated using the methods employed for discrete reflections as described in our previous publication (Grubb *et al.*, 2021 ▸). The fans can be simulated from a simple assembly of rods [Fig. 6 ▸(*a*) and 6 ▸(*b*)]. To simulate the propellor, the rods used in Fig. 6 ▸(*a*) need to be mixed with discs (projected spheres) [Figs. 6 ▸(*c*)–6 ▸(*e*)]. As will be shown Section 4.2, these two populations of objects sufficiently explain the observed CDS. To obtain the diamond pattern, the population of discs was replaced by ellipsoids [Figs. 6 ▸(*f*)–6 ▸(*h*)].

One of the differences between the fan and diamond patterns is the rate at which the intensity falls off with scattering angle. Although the central scattering peak in a diamond pattern appears to narrow at higher angles along the equator, the longitudinal width of the peak actually increases with the scattering vector, as in the fan-like pattern (Grubb *et al.*, 1991 ▸; Grubb & Prasad, 1992 ▸; Murthy *et al.*, 1996 ▸; Yang *et al.*, 1997 ▸). The rate of this increase is determined by the orientation of the scattering objects, which can be either voids or surfaces, and the width extrapolated to the meridian is determined by the length of the scattering entity. These orientation values are consistent with those obtained from WAXS data, and the length of the scattering entities is consistent with the estimates made from the lamellar reflections (Murthy *et al.*, 1996 ▸).

## 4. Elliptical features in small-angle scattering patterns

**Figure 7:**
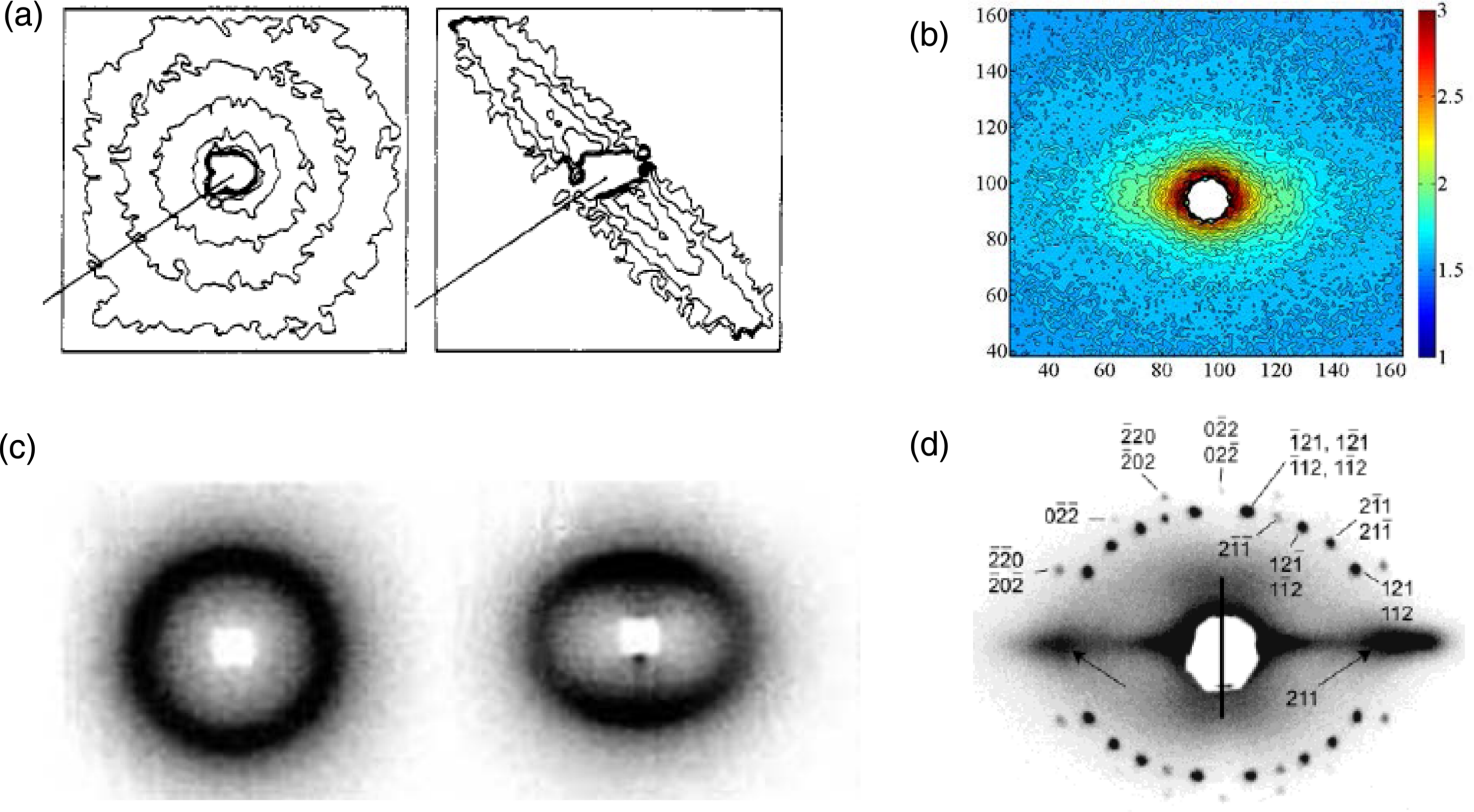
Elliptical patterns in various systems. (*a*) SANS pattern of polystyrene chains; reprinted from Hadziioannou *et al.* (1982 ▸) with permission from the American Chemical Society. (*b*) SAXS patterns of hair soaked in coconut oil and then heat-treated for 90 s at 180°C; adapted from Kamath *et al.* (2014 ▸) with permission from the Society of Cosmetic Chemists. (*c*) Polyurethane; adapted from Blundell *et al.* (2002 ▸) with permission from Elsevier. (*d*) Silica framework; reprinted from Hayward *et al.* (2004 ▸) with permission from the American Chemical Society.

An important observation that can be made in the SAXS patterns, both observed (Fig. 1 ▸) and simulated (Figs. 3 ▸ and 6 ▸), in the discrete reflections and in the CDS, is that there are features in the pattern that appear elliptical (Mildner, 1983 ▸; Brandt & Ruland, 1996 ▸). Although this has been reported in the literature, it has not been explored in depth. Some examples from the published literature are shown in Fig. 7 ▸. The most obvious example is the pattern from a stretched amorphous polystyrene [Fig. 7 ▸(*a*)] (Hadziioannou *et al.*, 1982 ▸); these are SANS data from atactic polystyrene using 95% protonated and 5% completely deuterated chains to examine the scattering from isolated chains. Similar scattering has been observed in SAXS from polymer melts under shear (Somani *et al.*, 2002 ▸). Ellipticity is equally obvious in the CDS from fibers with internal structure and voids, as seen in a SAXS pattern from hair [Fig. 7 ▸(*b*)]. Elliptical scattering is also observed when orientational correlations are present in an assembly of thin disc-like laponite particles in clay suspension that show discotic ordering (Lemaire *et al.*, 2002 ▸). Examples of elliptical patterns in discrete reflections are seen for stretched block copolymers (Brandt & Ruland, 1996 ▸) and polyurethanes [Fig. 7 ▸(*c*)] (Blundell *et al.*, 2002 ▸). The distribution of scattering domains in unstretched block copolymers is isotropic and random, and so the scattering is also isotropic along a circular track. When the sample is stretched, the domains move apart in the stretching direction as they come closer in the lateral direction. This affine deformation is the most natural explanation for the elliptical scattering from an oriented material. Finally, anisotropic shrinkage of the mesostructured silica film that occurs perpendicular to the substrate during drying, cross-linking and densification of the silica framework distributes the reflections along an elliptical trajectory [Fig. 7 ▸(*d*)] (Hayward *et al.*, 2004 ▸).

Though the elliptical form is apparent in the patterns shown in Fig. 7 ▸, it is not so obvious in the patterns shown in Figs. 1 ▸–3 ▸ ▸ and 6 ▸. But elliptical features are also present in these other patterns including lamellar reflections, as will be discussed in the following section.

### 4.1. Ellipticity in lamellar reflection

**Figure 8:**
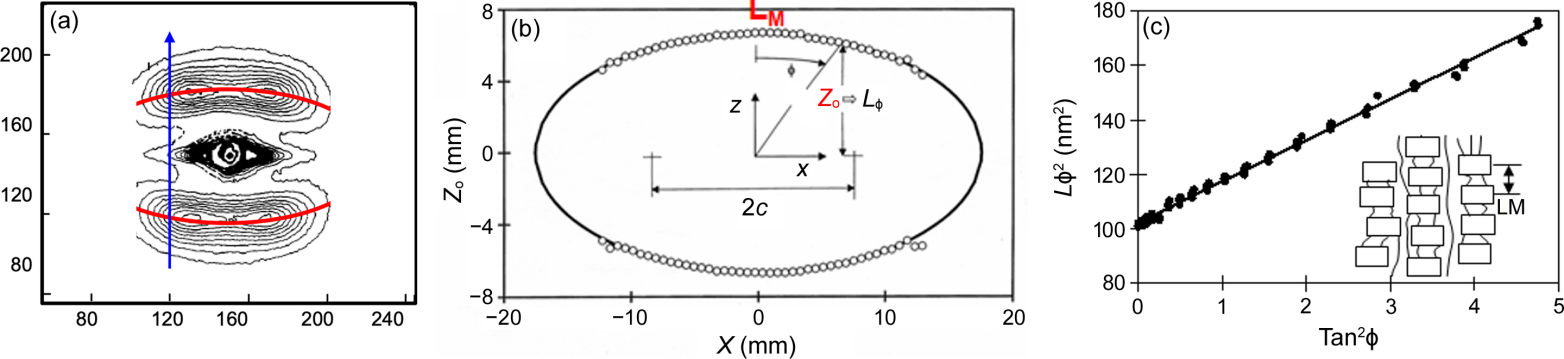
Elliptical trajectory of the lamellar reflections (Murthy *et al.*, 2000 ▸). (*a*) 2D SAXS pattern from an oriented nylon-6 fiber. (*b*) Plot of the lamellar peak position as a function of the distance perpendicular to the fiber axis in a drawn and annealed fiber. (*c*) Plot of 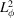 versus tan^2^ϕ.

The peak intensities of the discrete reflections shown in Figs. 1 ▸(*d*) and 1 ▸(*e*), 2 ▸(*a*)–2 ▸(*d*), and 3 ▸(*h*) and 3 ▸(*i*) do not lie on a layer line, or on the arc of a circle, but follow an elliptical trajectory. This can be seen by first analyzing the pattern as a series of *z* slices [Fig. 8 ▸(*a*)] and then plotting the positions of the peak maxima of the lamellar reflections [Fig. 8 ▸(*b*)]. The peak maxima fall on an ellipse out to ϕ angles as high as 75°. This elliptical trajectory can be further confirmed by linearizing the expression for an ellipse:
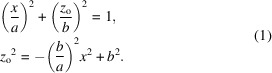
If the contour is an ellipse, the plot 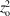 as a function of *x*^2^ should be a straight line. For convenience in extracting the lamellar parameters, this equation can be rearranged to
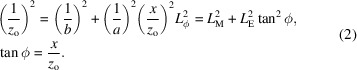
When (1/*z*_o_)^2^ is plotted as a function of (*x*/*z*_o_)^2^ = tan^2^ϕ, the intercept *L*_M_ gives the lamellar spacing along the draw direction (meridional axis), and the slope *L*_E_ is the fictitious spacing of the lamellae oriented along the equator (Murthy *et al.*, 2000 ▸). Fig. 8 ▸(*c*), which is typical of many polymers investigated in our laboratory, exhibits a linear fit up to 65° in ϕ, showing that the contour is very close to being an ellipse. This method necessarily fails at the equator where the *z* slice is parallel to the contour of the reflection. Finding the peak trajectory as a locus of minimum curvature of the intensity surface extends the elliptical fitting to even higher angles (Murthy *et al.*, 2000 ▸; Grubb *et al.*, 2016 ▸).

The trajectory for the bar pattern is a single ellipse, but for the eyebrow and butterfly patterns each requires two ellipses (Fig. 3 ▸). This has been extensively discussed in our earlier publications (Grubb *et al.*, 2021 ▸; Wang *et al.*, 2007 ▸).

An elliptical trajectory is a natural consequence of the affine deformation of a lattice, such as those of block copolymers. But the observed changes in lamellar spacing in many samples are not consistent with such affine deformation. In the case of semicrystalline polymers and liquid crystals, it is not necessary to have affine transformation of the domains to obtain an elliptical trajectory. Lamellae in a semicrystalline polymer or a liquid crystal could move apart along the orientation direction as they move closer together in the lateral direction. A moderate amount of disorder, such as randomly placed lamellar stacks that are small and spread the reflection in the lateral direction combined with a range of lamellar tilts and a range of stack axis directions, can make the peak positions trace out an ellipse without the requirement of an affinely deformed lattice.

### 4.2. Central diffuse scattering and the equatorial streak

**Figure 9:**
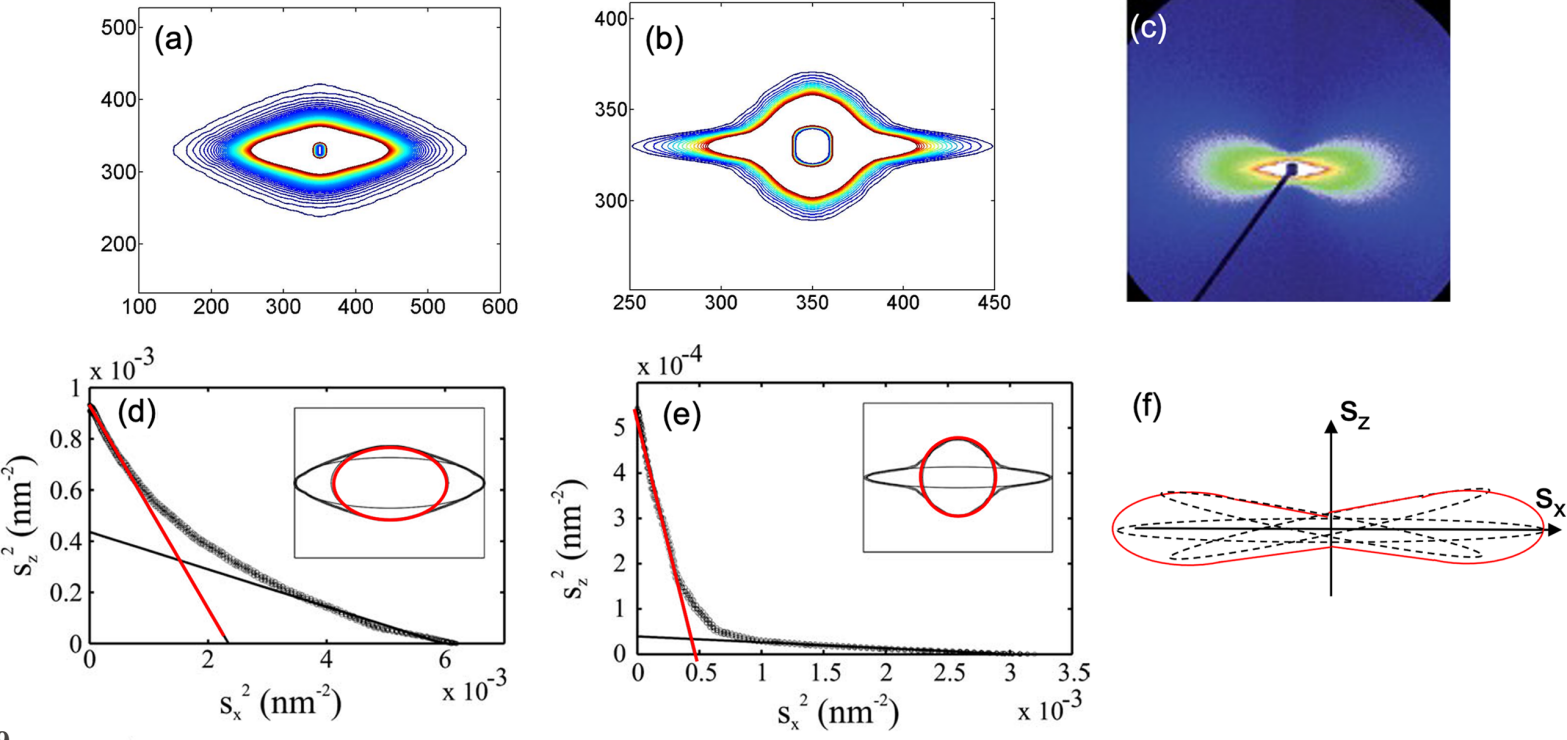
(*a*) Diamond-shaped SAXS pattern from a solution-spun polyacrylo­nitrile (PAN). (*b*) Propeller-shaped pattern from a gel-spun PAN fiber. (*c*) Fan-shaped diffraction pattern from micro-voids in oriented bundles of γ-irradiated polyacrylo­nitrile-based carbon fibers. Linear fitting of the linearized intensity plot for (*d*) the diamond-like pattern shown in (*a*), and a superposition of two implied ellipses (*e*) for the propeller-like pattern shown in (*b*). (*f*) Illustration of how the misorientation-dominated fan pattern arises. In these figures, *S_x_* and *S_z_* correspond to *x* and *z*_o_ in equations (1) and (2) and Fig. 8 ▸. (*a*), (*b*), (*d*) and (*e*) Reproduced from Wang *et al.* (2012 ▸). (*c*) Reproduced from Feng *et al.* (2018 ▸) with permission from the Royal Society of Chemistry.

In addition to the two- and four-point reflections, CDS with an oval, diamond or two-bladed propeller shape also has elliptical characteristics. There are in general five types of CDS: isotropic, oval, fan, diamond and propeller. While simple anisotropic scattering can be fitted to a single ellipse, ellipticity in other types of CDS can be demonstrated by linearizing the equation for the ellipses [equations (1) and (2)]. The results are shown in Fig. 9 ▸. If in Figs. 9 ▸(*a*) and 9 ▸(*b*) there was only one ellipse, then there should be a single straight line in the plot 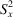 versus *x*^2^. Instead, there are two straight-line segments in the data for diamond- and propeller-shaped patterns, indicating that there are two components [Figs. 9 ▸(*d*) and 9 ▸(*e*)], one nearly isotropic central scattering and the other the anisotropic streak (Wang *et al.*, 2012 ▸). In the case of a fan-like pattern that is dominated by misorientation [Fig. 9 ▸(*c*)] (Feng *et al.*, 2018 ▸), the outside edges of the fan can be fitted to an ellipse to obtain the degree of orientation from the ellipticity of the fitted curve as shown in Fig. 9 ▸(*f*) (Wang *et al.*, 2012 ▸). Thus, the changes in the central scattering can be completely reconstructed using a two-component model.

### 4.3. Utility of the elliptical features

The elliptical features in the scattering pattern contain details of the deformation that gives rise to anisotropy. In some instances, there could be correlation between longitudinal and transverse deformation (affine transformation). In others, it could be due to the random placement of the lamellar stacks modified by chain slip and stack rotation or interlamellar shear. For example, the three parameters that describe the elliptical trajectory in semicrystalline polymers, the lamellar spacing, chain slip and lamellar slip, are related to the interaction of the lamellae with the surrounding amorphous regions and fibrils. Elliptical features are also useful in providing a framework or constraint for analyzing the scattering patterns. This will be discussed in the following section.

## 5. Parameterization of the pattern

Section 3 dealt with the analysis of the SAXS pattern by modeling the structure using known principles of lamellar assembly. This approach was validated by demonstrating the agreement of the patterns simulated from models with the observed diffraction patterns. Currently available computational tools allow these microstructures to be rapidly refined. The problem with a modeling approach is that the model derived from a pattern is not unique. An alternative approach is to parameterize the data. The parameters derived from such functional fitting can be assigned to features in known and validated structural models, structures validated by simulating scattering that agrees with the observed diffraction patterns or by complementary techniques such as microscopy. In this latter approach, the pattern can be quickly reduced to a few important, reliable parameters that can be used for quantitative comparison of the changes that occur during testing and processing. In most instances, these are sufficient to understand the changes in the key aspects of the structure such as fibril size and orientation and lamellar spacing, stack size and orientation.

**Figure 10:**
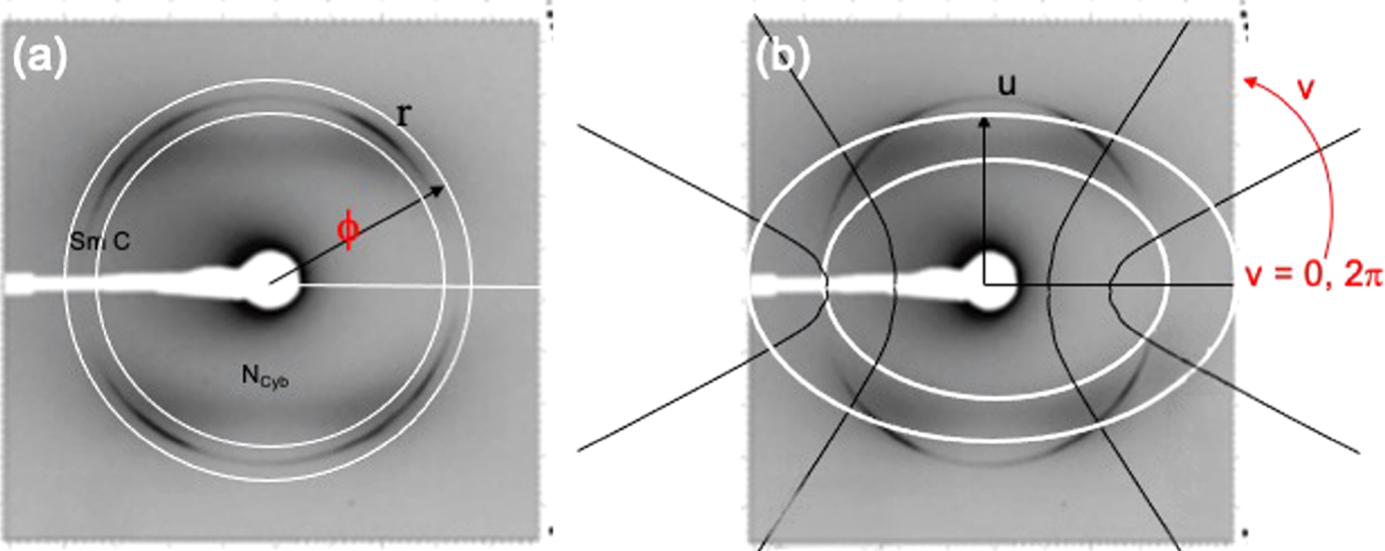
Description of the elliptical scheme, adopted from Francescangeli *et al.* (2011 ▸) with permission from the American Physical Society. (*a*) Polar coordinates that can be used to fit the sharp smectic reflections. (*b*) Elliptical coordinates used to fit the weak and diffuse four-point eyebrow pattern.

Patterns such as isotropic diffuse scattering from orientationally disordered anisotropic structures or the discrete reflections in a smectic phase that fall on a circle [Fig. 10 ▸(*a*)] can be best described in polar coordinates. But the reflections from a cybotactic nematic phase are curved along an elliptical arc [weak and diffuse reflections in Fig. 10 ▸(*b*)]. Therefore, the intensity in such patterns can be best described in elliptical coordinates (*u*, *v*) using two orthogonal functions, *f*(*u*) and *g*(*v*). The coordinate system is schematically illustrated in Fig. 10 ▸(*b*). Moving along the *u* coordinate is analogous to going along *r* in polar coordinates and increases the size of the ellipse. Going along *v* is moving along the ellipse, analogous to changing ϕ. The observed pattern can be reconstructed with the least number of parameters by expressing the intensity in elliptical coordinates as a function of *f*(*u*) and *g*(*v*): *f*(*u*) along the hyperbola and *g*(*v*) along the ellipse.

**Figure 11:**
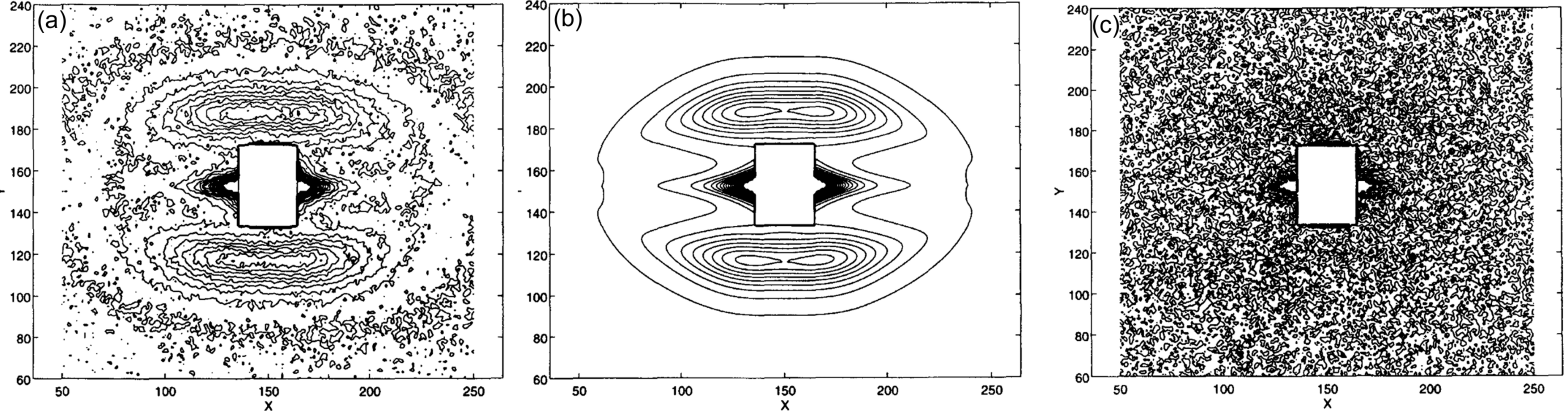
Example of complete SAXS pattern fitting, both the discrete lamellar reflections and the equatorial streak. These are contour maps of the intensity distribution. The intensity of 30–350 counts is divided into 20 contours. The *x* and *y* axes are marked in channel numbers. (*a*) Observed data. (*b*) Fitted data. (*c*) Difference map with contour levels between 20 and 50 counts (Murthy *et al.*, 1997 ▸).

Using these ideas, different types of two- and four-point patterns, as well as CDS including the equatorial streak, can be fitted to functions with only five parameters, two for the *u*–*v* position and two for the widths (Δ*u* and Δ*v*) of the reflections, and a fifth parameter for the ellipticity of the pattern (Murthy *et al.*, 1997 ▸). Fig. 11 ▸ shows a complete fit of both the discrete reflections and the equatorial streaks. There is good agreement between the fitted contours and the observed data. These results show that (1) the trajectory of the lamellar SAXS scattering is neither straight nor circular; (2) the reflections are curved because of randomly placed lamellar stacks with a range of tilts and rotations; (3) this curvature cannot be efficiently handled in Cartesian coordinates. The elliptical features of the SAXS patterns, including the equatorial streak, suggest that the entire SAXS pattern can be optimally fitted in elliptical coordinates with the least number of parameters. Following the changes in the central diffuse scattering and the lamellar diffraction pattern during deformation allowed us to show the extent to which these structures determine the mechanical properties of the polymer (Murthy & Grubb, 2002 ▸, 2003 ▸; Wang *et al.*, 2009 ▸).

## 6. Conclusions

The two features in SAXS often have elliptical shapes that can be used to our advantage to efficiently analyze these patterns. The two components in the equatorial streak, one isotropic and the other oriented, can be analyzed to obtain the size and orientation of voids, fibrils or surfaces. Ellipticity in the central diffuse scattering can be attributed to the affine deformation induced by flow or stretching. The two-point banana, four-point eyebrow and four-point butterfly patterns can be simulated from a random assembly of lamellar stacks modified by chain slip and stack rotation or interlamellar shear. These can also be analyzed in elliptical coordinates. Thus, the whole 2D SAXS data set can be profile fitted efficiently in elliptical coordinates to fully characterize or featurize the SAXS diffraction pattern with the least number of parameters. Such rapid analyses of data from a large number of samples are required for implementing ML and AI methods in materials development.
